# Racial and Sex Differences in Genomic Profiling of Intrahepatic Cholangiocarcinoma

**DOI:** 10.1245/s10434-024-16141-8

**Published:** 2024-09-09

**Authors:** Diamantis I. Tsilimigras, Hunter Stecko, Ioannis Ntanasis-Stathopoulos, Timothy M. Pawlik

**Affiliations:** 1 Department of Surgery, Division of Surgical Oncology, The Ohio State University Wexner Medical Center and James Comprehensive Cancer Center, Columbus, OH USA; 2 Department of Clinical Therapeutics, National and Kapodistrian University of Athens, Athens, Greece

**Keywords:** Race, Sex, Gene, Genomic, Profiling, Intrahepatic cholangiocarcinoma, Next generation sequencing

## Abstract

**Background:**

Racial and sex disparities in the incidence and outcomes of patients with intrahepatic cholangiocarcinoma (iCCA) exist, yet potential genomic variations of iCCA based on race and sex that might be contributing to disparate outcomes have not been well studied.

**Methods:**

Data from the American Association for Cancer Research Project GENIE registry (version 15.0) were analyzed to assess genetic variations in iCCA. Adult patients (age >18 years) with histologically confirmed iCCA who underwent next-generation sequencing were included in the analytic cohort. Racial and sex variations in genomic profiling of iCCA were examined.

**Results:**

The study enrolled 1068 patients from 19 centers (White, 71.9%; Black, 5.1%; Asian, 8.4%, other, 14.6%). The male-to-female ratio was 1:1. The majority of the patients had primary tumors (73.7%), whereas 23.0% had metastatic disease sequenced. While *IDH1* mutations occurred more frequently in White versus Black patients (20.8% vs. 5.6%; *p* = 0.021), *FGFR2* mutations tended to be more common among Black versus White populations (27.8% vs. 16.1%; *p* = 0.08). Males were more likely to have *TP53* mutations than females (24.3% vs. 18.2%, *p* = 0.016), whereas females more frequently had *IDH1* (23.3% vs 16.0 %), *FGFR2* (21.0% vs. 11.3%), and *BAP1* (23.4% vs. 14.5%) mutations than males (all *p* < 0.05). Marked variations in the prevalence of other common genomic alterations in iCCA were noted across different races and sexes.

**Conclusion:**

Distinct genomic variations exist in iCCA across race and sex. Differences in mutational profiles of iCCA patients highlight the importance of including a diverse patient population in iCCA clinical trials as well as the importance of recognizing different genetic drivers that may be targetable to treat distinct patient cohorts.

**Supplementary Information:**

The online version contains supplementary material available at 10.1245/s10434-024-16141-8.

Intrahepatic cholangiocarcinoma (iCCA) is an aggressive tumor of the liver that originates from the intrahepatic bile ducts and comprises 5 to 10% of primary liver malignancies.^1,2^ The prognosis for iCCA patients remains poor, with 5-year survival ranging from 5 to 30% depending on the disease stage at diagnosis.^3^

Although surgery followed by adjuvant chemotherapy is the recommended first-line therapy for patients with resectable tumors,^4^ approximately 60 to 70% of patients with iCCA present with unresectable tumors or extrahepatic disease at the time of diagnosis.^5^ For these patients, a combination of gemcitabine plus cisplatin (GemCis) plus durvalumab or pembrolizumab is recommended as first-line therapy,^6^ whereas patients who progress on first-line therapy usually receive fluorouracil, leucovorin, and oxaliplatin (FOLFOX) as second-line therapy.^5,7^ However, the efficacy of second-line therapy is modest, with an objective response of 5% and a median overall survival of 6.2 months.^7^ As such, studies have focused recently on the identification of potentially actionable genetic alterations to develop effective targeted therapies for patients who have iCCA with specific molecular profiles.

Molecular profiling of iCCA has allowed for a better understanding of the distinct genomic landscape characterized by various oncogenic drivers that contribute to the pathogenesis and progression of disease.^8^ Indeed, up to 50% of iCCA patients present with potentially actionable genomic alterations including *IDH1* (~20%), *ARID1A* (~20%), *BAP1* (~18%), *TP53* (~20%), and *FGFR2* (~15%) fusions.^8^ Among these, *IDH1* mutations and *FGFR2* fusions have been considered the most promising targets in iCCA treatment.^5,8^ Recently, *FGFR* and *IDH1* inhibitors received Food and Drug Administration (FDA) approval and currently are recommended for iCCA patients with *FGFR2* fusions/rearrangements and *IDH1* mutations, respectively.^4^

Despite the encouraging results from precision oncologic studies, enrollment of Black and other non-White patients has been relatively low,^8,9^ thereby limiting our comprehensive understanding of the genomic landscape of iCCA in diverse populations. In turn, racial variations in the genomic profiling of iCCA may exist, but have not been studied to date. In addition, racial and sex disparities in the incidence and outcomes of patients with iCCA have previously been reported,^10^ yet the contribution of genetic variations to disparate outcomes of iCCA remains unknown. To this end, the current study aimed to assess comprehensively the genomic profile of iCCA using a large dataset of iCCA patients who had next-generation sequencing (NGS) of their tumors. Specifically, we sought to assess genomic alterations based on race and sex that could be related to differences in response to treatment and prognosis among a diverse patient population with iCCA.

## Methods

### Study Cohort and Dataset

Data were derived from the American Association of Cancer Research (AACR) Project Genomics Evidence of Neoplasia Information Exchange (GENIE) dataset (v15.0, released 27 December 2023) through cBioPortal.^11^ The GENIE database was initially launched in 2017 and currently includes somatic genomic data of approximately 167,000 patients from 19 participating institutions.^11^

All institutions use NGS assays to detect somatic mutations in formalin-fixed paraffin-embedded tumor samples using targeted panels of varying gene numbers. Structural variants (e.g., fusions) as well as copy number alterations (e.g., amplifications or deletions) also are provided for a subset of patients. Further details on the methodology used in each institution are available in the GENIE cohort data guide.^11^

Adult patients with a diagnosis of iCCA who had available NGS data were identified and included in the analytic cohort. Data from the OncoKB database, provided by cBioPortal, were used to determine pathogenic gene alterations, as previously described.^12,13^ Germline mutations were excluded from the analysis. Genomic variations based on race and sex of participants were examined. Because all data originated from a publicly available de-identified database, the current study was deemed exempt from the Institutional Review Board of the Ohio State University.

### Statistical Analysis

Data are expressed as median (interquartile range [IQR]) for continuous variables and as frequency (%) for categorical variables. The prevalence of the most frequent genomic alterations (including somatic mutations, structural variants, and copy number alterations) among patients with iCCA was calculated. Differences in frequency of genomic alterations relative to race, sex, and primary versus metastatic tumors were examined with the use of chi-square or Fisher’s exact tests. The significance levels of co-occurrence and mutual exclusivity for a pair of variant genes were calculated by the Mutual Exclusivity Modules statistical method from cBioportal.^14^

## Results

### Patient and Sample Characteristics

The study identified 1068 patients who underwent genomic profiling of iCCA and included these patients in the analytic cohort. The median age at the time of tumor sequencing was 64 years (IQR, 55–71 years). The majority of the patients were White (*n* = 768, 71.9%) followed by Asian (*n* = 90, 8.4%) and Black (*n* = 54, 5.1%) patients. A subset of individuals self-identified as “other/unknown” (*n* = 156, 14.6%). The male-to-female ratio was 1:1 (532 males [49.8%] and 533 females [49.9%], 3 of unknown sex [0.3%]) (Table 1).

**Table 1:** Characteristics of patients who underwent NGS of iCCA

	Total patients (*n* = 1068) *n* (%)
Age, years	64 (IQR 55–71)
Sex	
Male	532 (49.8)
Female	533 (49.9)
Unknown	3 (0.3)
Race	
White	768 (71.9)
Asian	90 (8.4)
Black	54 (5.1)
Unknown/other	156 (14.6)
No samples/patient	
1	990 (92.7)
≥2	78 (7.3)
Sample type (*n* = 1108)	
Primary tumor	817 (73.7)
Metastasis	255 (23.0)
Unknown	36 (3.3)

NGS, next-generation sequencing; iCCA, intrahepatic cholangiocarcinoma

The vast majority of the patients (*n* = 990, 92.7%) had one tumor sample sequenced, whereas 7.3% (*n* = 78) had two or more tumor samples analyzed (total number of samples, 1108). Most samples were derived from the primary tumor (*n* = 817, 73.7%), whereas the remaining samples were obtained from metastatic tumors (*n* = 255, 23.0%) or otherwise unspecified samples (*n* = 36, 3.3%).

### Genomic Profiling of iCCA: Somatic Mutations, Structural Variants, and Copy Number Alterations

**Fig. 1:**
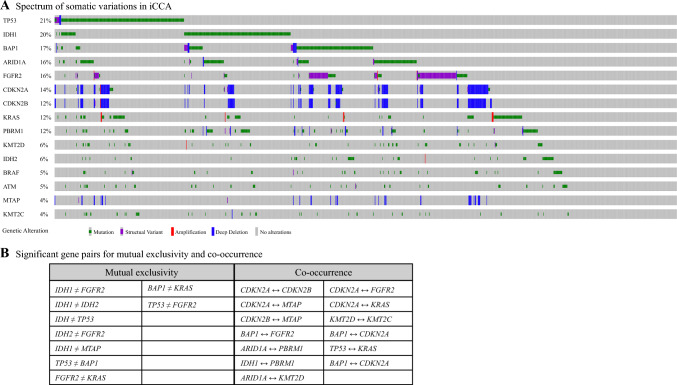
**a** Oncoprint showing the most commonly mutated genes among patients with intrahepatic cholangiocarcinoma (iCCA) together with their respective genetic alterations. **b** Patterns of significant gene pairs for co-occurrence and mutual exclusivity.

Overall, the most commonly mutated genes among all iCCA patients were *TP53* (21%), *IDH1* (20%), *BAP1* (17%), *ARID1A* (16%), *FGFR2* (16%), *CDKN2A* (14%), *CDKN2B* (12%), *KRAS* (12%), and *PBRM1* (12%). The exact genomic alterations for the most commonly mutated genes are noted in Fig. 1a. Notably, *IDH1/2* and *FGFR2* alterations were mutually exclusive. The *FGFR2* gene was frequently co-mutated with *BAP1*, whereas the *IDH1* gene was commonly co-mutated with *PBRM1* (all *p* < 0.05; Fig. 1b).

When examining the exact genomic alterations among the iCCA samples, point mutations were most frequently observed in *IDH1* (19.9%), *TP53* (19.8%), *BAP1* (16.5%), and *ARID1A* (16.4%) genes. All *IDH1* mutations were missense mutations (100%), with the overwhelming majority representing single-nucleotide variations at R132 in exon 4 (99.0%, 207/209). Notably, *IDH1*-mutated iCCA also frequently had concurrent *ARID1A* (21.8%), *PBRM1* (18.8%), and *BAP1* (12.9%) mutations. While *FGFR2* point mutations were noted in only 5.1% of the iCCA samples, the prevalence of *FGFR2* structural variations (including *FGFR2* fusions/re-arrangements) was 12.5% (122 of 978 samples). Samples of iCCA with *FGFR2* fusions/re-arrangements also were noted to have co-mutations in *BAP1* (32.0%) and *KMT2D* (8.2%) genes. The incidence of concurrent *FGFR2* point mutations and fusions/rearrangements was 4.9%.

Copy number alterations also were common among iCCA samples. The study observed *CDKN2A* homologous deletions in 14.9% (132/886) of samples followed by homologous deletions in *CDKN2B* (13.8%, 122/885) and *MTAP* (12.8%, 37/289) genes (Table 2).

**Table 2:** Most common point mutations, structural variants, and copy number alterations among iCCA samples

	Top 20 genes with point mutations		
Gene	Mutation *n*	Total *n*	Frequency %
IDH1	220	1108	19.9
TP53	219	1107	19.8
BAP1	169	1022	16.5
ARID1A	167	1021	16.4
KRAS	129	1108	11.6
PBRM1	111	966	11.5
KMT2D	60	968	6.2
KMT2C	48	805	6.0
IDH2	62	1087	5.7
BRAF	58	1108	5.2
ATM	56	1085	5.2
FGFR2	57	1108	5.1
TERT	49	961	5.1
PIK3CA	53	1108	4.8
RASA1	35	846	4.1
NF1	40	1044	3.8
SMAD4	38	1105	3.4
FAT1	30	899	3.3
NRAS	35	1108	3.2
BRCA2	31	1043	3.0

	Top 5 genes with structural variants		
Gene	Mutation *n*	Total *n*	Frequency %
FGFR2	122	978	12.5
BICC1	38	978	3.9
BAP1	10	978	1.0
TP53	8	978	0.8
PBRM1	7	954	0.7

	Top 5 genes with copy number alterations		
Gene	Mutation *n*	Total *n*	Frequency %
CDKN2A (homdel)	132	886	14.9
CDKN2B (homdel)	122	885	13.8
MTAP (homdel)	37	289	12.8
MDM2 (amp)	28	886	3.2
MCL1 (amp)	27	857	3.2

iCCA, intrahepatic cholangiocarcinoma

### Genomic Alterations Among Patients With Primary Versus Metastatic iCCA

Among the patients with primary iCCA, the most common genomic alterations were *BAP1* (20.6%), *TP53* (20.4%), *IDH1* (19.0%), *FGFR2* (18.1%), and *CDKN2A* (17.3%). In contrast, the most prevalent genomic variations among individuals with metastatic iCCA were *TP53* (23.5%), *IDH1* (21.0%), *ARID1A* (17.6%), *KRAS* (15.1%), and *PBRM1* (14.7%). Alterations in *BAP1* (20.6 vs. 14.1%; *p* = 0.036), *CDKN2A* (17.3 vs. 10.4%; *p* = 0.009), and *FGFR2* (18.1 vs. 9.9%, *p* = 0.002) genes were more frequent among patients with primary versus metastatic iCCA, whereas alterations in *SMAD4* (3.0 vs. 6.8%; *p* = 0.013) were more prevalent among patients with metastatic tumors (Table S1).

### Racial and Sex Variations in Genomic Profile of iCCA

**Fig. 2:**
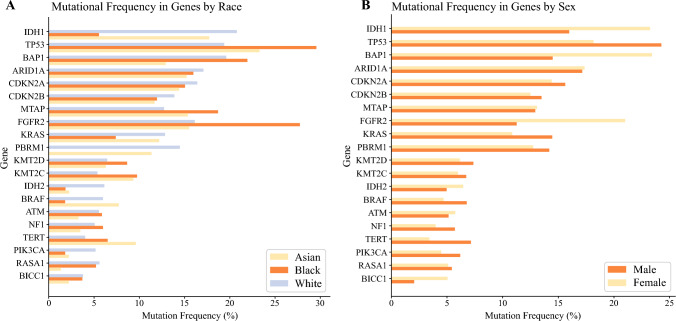
Variations in mutational frequency of genes by **a** race and **b** sex among patients with iCCA.

A subsequent analysis was performed to examine genomic variations of iCCA based on race. The most prevalent genomic alterations of any type among White patients were *IDH1* (20.8%) and *BAP1* (19.6%) mutations, whereas among Asian individuals, *TP53* (23.3%) and *IDH1* (17.8%) mutations were the most common genomic alterations. In contrast, Black patients most commonly had alterations in *TP53* (29.6%) followed by *FGFR2* (27.8%) genes. Comparison of the three groups showed that *IDH1* mutations were more frequently enriched in White (20.8%) versus Asian (17.8%) or Black (5.6%) patients (*p* = 0.021). Similarly, *PBRM1* mutations were more prevalent among White (14.5%) versus Asian (11.4%) or Black (0%) individuals (*p* = 0.015). In contrast, *FGFR2* tended to be more frequently mutated among Black individuals (27.8%) compared with Asian (15.6%) or White (16.1%) individuals, yet the difference did not reach statistical significance (*p* = 0.08). Marked variations in the prevalence of other common genetic mutations in iCCA were noted across different races (Table S2, Fig. 2a).

Significant genomic variations also were observed based on the sex of the iCCA patients. Among the female patients, the most prevalent alterations were *BAP1* (23.4%) and *IDH1* (23.3%) mutations, whereas men most frequently harbored mutations in *TP53* (24.3%) and *ARID1A* (17.2%) genes. Comparison of the two groups showed that *TP53* mutations were more likely to occur in males than females (24.3 vs. 18.2%; *p* = 0.016), whereas *IDH1* mutations were more prevalent in females (23.3 vs. 16.0%; *p* = 0.003). In addition, alterations in *FGFR2* (21.0 vs. 11.3%) and *BAP1* (23.4 vs. 14.5%) genes were more frequent among females than males (both *p* < 0.001). Marked variations in the prevalence of other genomic alterations were noted across sexes (Table S3, Fig. 2b).

## Discussion

Molecular characterization of iCCA can identify potentially actionable genomic alterations that may lead to the development of effective targeted therapies and improve the prognosis of patients with advanced iCCA.^5,15^ Despite the increasing incidence of iCCA among Asian and Black patients, these patient populations have been largely underrepresented in existing precision oncology studies.^10,16^ In turn, racial variations in molecular profiling of iCCA may exist but have not been studied to date.

The current study was important because it specifically examined the genomic profile of iCCA, as well as the racial and sex variations in the molecular profiling of iCCA. Overall, the most commonly mutated genes among all the iCCA patients were *TP53* (21%), *IDH1* (20%), *BAP1* (17%), *ARID1A* (16%), and *FGFR2* (16%). While *IDH1* mutations were more commonly observed among White individuals, *FGFR2* tended to be more frequently mutated among Black individuals with iCCA. While *TP53* mutations were more common among males, *IDH1* and *FGFR2* alterations were more frequent among female patients with iCCA. Marked variations in other frequently mutated genes were noted according to race and sex of the patients with iCCA. To the best of our knowledge, this is the largest study to report the prevalence of somatic genomic alterations among patients with iCCA and the first to examine racial and sex variations in the genomic profiling of iCCA.

During the past decade, significant advances have been realized in the molecular characterization of biliary tract cancers including iCCA.^5,8,9^ Next-generation sequencing technologies have identified genetic aberrations thought to contribute to tumorigenesis and cancer progression in iCCA.^8,15^ The literature suggests that up to 50% of iCCA patients may present with at least one potentially actionable genetic alteration.^8^ Although previous clinical trials enrolled and analyzed patients with biliary tract cancers (i.e., iCCA, extrahepatic cholangiocarcinomas, gallbladder cancers) all together,^17,18^ recent evidence suggests that iCCA has a distinct biologic profile compared with other tumors.^8^ In analyzing 195 patients with intra- and extra-hepatic cholangiocarcinomas, Lowery et al.^8^ demonstrated that mutations in *IDH1* and *TP53* as well as *FGFR2* fusions were more commonly seen in iCCA, whereas *KRAS*, *SMAD4,* and *STK11* alterations more frequently occurred in extrahepatic cholangiocarcinomas. The data suggested that iCCA is a biologically unique disease with distinct molecular targets compared with other biliary tract cancers.^8^ These findings can have important implications as biomarker-driven clinical trials have shown a clear survival benefit for patients with advanced biliary tract cancers who received matched molecular targeted agents.^9^

Current National Comprehensive Cancer Network (NCCN) guidelines recommend molecular testing for unresectable or metastatic iCCA that should include *IDH1* mutations, *FGFR2* fusions or rearrangements, *ERBB2* amplifications, *BRAF* V600E mutations, and *RET* gene fusions as well as tumor mutational burden (TMB) and microsatellite instability (MSI).^4^ The current study demonstrated that the most commonly mutated genes among all iCCA patients were *TP53* (21%), *IDH1* (20%), *BAP1* (17%), *ARID1A* (16%), and *FGFR2* (16%), whereas the mutations in *KRAS* (12), *IDH2* (6%), and *BRAF* (5%) genes were less frequently observed. Among these genomic alterations, *IDH1* mutations and *FGFR2* fusions have been considered the most promising targets in iCCA.^5,9^ Both *IDH* 1 and 2 are integral enzymes for cellular respiration that reduce NADPH to NADP+ during the conversion of isocitrate to ketoglutarate in the citric acid cycle.^19,20^

Mutations in *IDH1*/2 lead to accumulation of the oncometabolite 2-hydroxyglutarate (2-MG), inducing epigenetic changes in the tumors such as DNA hypermethylation and altered expression of chromatin remodelers.^19–21^ On the other hand, *FGFR* alterations, most commonly *FGFR2* fusions, are responsible for the activation of various downstream-signaling pathways, including RAS/RAF/MEK, JAK/STAT, and PI3L/AKT pathways, which contribute to malignant transformation and cancer progression.^22^

Recent studies have demonstrated that patients harboring *FGFR2* fusions exhibit substantial sensitivity to *FGFR* inhibitors. For example, a multicenter phase 2 study (FIGHT-202) noted that among individuals with locally advanced or metastatic iCCA with *FGFR2* fusions/rearrangements, pemigatinib was associated with an objective response rate of 35.5% and a median progression-free survival of 6.9 months, which were superior to those of individuals with iCCA harboring other FGF/FGFR alterations or no FGF/FGFR2 alterations.^23^ A﻿nothe﻿r phase 3 randomized controlled clinical trial (ClarIDHy) demonstrated that patients with chemotherapy-refractory cholangiocarcinoma with *IDH1* mutations who received ivosidenib (an *IDH1* inhibitor) had a median PFS of 2.7 months versus 1.4 months among patients who received placebo (*p* < 0.001).^24^ In contrast, among patients with surgically treated iCCA, the presence of *KRAS* (particularly G12 *KRAS* variant) mutations predicted worse overall (hazard ratio [HR], 1.69, 95% confidence interval [CI 1.31–2.18) and disease-free survival (HR, 1.47; 95% CI 1.16–1.88) than wild-type *KRAS* individuals.^25^ Similarly, *TP53* mutations have been generally associated with poor prognosis and limited response to conventional chemotherapy among patients with iCCA.^26^ These data highlight the need for comprehensive genomic profiling in iCCA to tailor therapeutic strategies and optimize outcomes for patients with iCCA.

To date, *IDH1* inhibitor ivosidenib and *FGFR2* inhibitors (i.e., pemigatinib, futibatinib) have received FDA approval for the treatment of patients with advanced, refractory metastatic cholangiocarcinoma with *IDH* mutations and *FGFR2* fusions/rearrangements, respectively.^4,5^ Previously, *FGFR2* translocations have been reported as mutually exclusive with *IDH1* mutations,^8^ a finding confirmed by this study. The current study also demonstrated that *FGFR2* alterations frequently co-occurred with both *BAP1* mutations and *CDKN2A* deletions, whereas *IDH1* mutations co-occurred with *PBRM1* mutations. This finding requires further investigation because it might explain why not all patients with *FGFR2* fusions and *IDH1* mutations respond to *FGFR2* and *IDH1* inhibitor therapy, respectively. In light of recent advances in personalized treatments with combination therapies,^27^ these data suggest that a combination of targeted agents might be recommended to patients with molecularly complex cancers, including iCCA.

Marked disparities in the enrollment of minority populations in precision oncology studies have been noted, with racial/ethnic minority populations disproportionally underrepresented in clinical trials relative to their cancer incidence in the U.S. population.^16^ In the current study, the vast majority of patients who underwent NSG of their iCCA were White (71.9%), whereas Black individuals comprised only 5.1% of the cohort. This percentage was lower than the percentage of Black individuals with iCCA diagnosed between 2004 and 2015 in the United States (~8.4%).^2^ Notably, the current study demonstrated that *IDH1* mutations were more frequently identified among White (20.8%) versus Asian (17.8%) or Black patients (5.6%), whereas *FGFR2* alterations were more frequently observed among Black individuals (27.8%). These data are important because up to one third of Black patients might be candidates for targeted therapies, a proportion markedly higher compared with the enrollment of Black individuals in landmark clinical trials investigating *FGFR2* inhibitors (range, 4–8%).^23,28,29^ In addition, *IDH1* and *FGFR2* alterations were more frequent among female versus male patients, suggesting that these patients are more likely to benefit from targeted therapies, and future clinical trials could be more enriched with these particular populations. Collectively, the data demonstrated that only a low number of non-White patients had undergone sequencing of iCCA, with marked variations in the genomic profiling of their tumors, highlighting the importance of mutation analysis among all patients with iCCA irrespective of their race and sex. Efforts to enroll a larger number of non-White patients with iCCA in precision oncology studies are needed to help address racial disparities, aid in future clinical trial design, and ideally lead to better insights into the reasons for differences in disease-specific outcomes among diverse populations with iCCA.^10^

One of the strengths of the current study was the analysis of a large number of patients with genomic data derived from multiple academic institutions. Nevertheless, certain limitations should be taken into consideration when the results are interpreted. Genomic data were aggregated from multiple academic centers, each using different NGS assays with different gene panel sets and tumor content criteria.^11^ The use of the cBioPortal platform, however, allowed us to retrieve the actual number of patients tested for each gene (i.e., correct denominator) and to calculate mutational frequency of certain genes accurately.^14^ In addition, the GENIE database lacked detailed information regarding patient-level characteristics including comorbidities, obesity, smoking, family history of cancer, thus the association of these with certain genomic profiles could not be assessed. Data on iCCA subtypes and microsatellite instability (MSI) status also were not available in the AACR GENIE database, so we were unable to investigate racial and sex genomic differences relative to iCCA subtypes and MSI status. Information on cancer stage, treatment characteristics, and survival were also not available, thus we were unable to assess the prognostic implications of certain genomic alterations among iCCA patients.

In conclusion, distinct genomic variations existed in iCCA across race and sex. *IDH1* mutations were most commonly identified in White patients with iCCA, whereas *FGFR2* fusions were more prevalent among Black individuals. *TP53* mutations were more common among males, whereas *IDH1* and *FGFR2* alterations were more frequent among female patients with iCCA. Differences in mutational profiles of iCCA patients highlight the importance of including a diverse patient population in iCCA clinical trials, as well as the significance of recognizing different genetic drivers that may be targetable to treat distinct patient cohorts.

## Supplementary Information

Below is the link to the electronic supplementary material.

Supplementary file1 (DOCX 22 kb)

## Data Availability

All data are publicly available from the CBioPortal platform upon request and acceptance by the AACR committee.
