# Supplementary material for: Racial and Sex Differences in Genomic Profiling of Intrahepatic Cholangiocarcinoma
Source: Ann Surg Oncol. 2024 Sep 9;31(13):9071–8. doi: 10.1245/s10434-024-16141-8 (PMC11549159; doi:10.1245/s10434-024-16141-8)
Supplement: Supplementary file 1 — Supplementary file1 (DOCX 22 kb) [file 10434_2024_16141_MOESM1_ESM.docx]

**Supplemental Table 1.** Genomic Alterations by Primary versus Metastatic iCCA

| **Gene** | **(A) Metastatic iCCA** | **(B) Primary iCCA** | **p-Value** | **Enriched in** |
| --- | --- | --- | --- | --- |
| TP53 | 59 (23.51%) | 162 (20.40%) | 0.289 | (A) Metastasis |
| IDH1 | 53 (21.03%) | 151 (19.02%) | 0.523 | (A) Metastasis |
| KRAS | 38 (15.08%) | 94 (11.84%) | 0.191 | (A) Metastasis |
| ARID1A | 36 (17.56%) | 131 (17.24%) | 0.917 | (A) Metastasis |
| BAP1 | 29 (14.15%) | 157 (20.63%) | 0.0364 | (B) Primary |
| PBRM1 | 27 (14.75%) | 95 (13.07%) | 0.545 | (A) Metastasis |
| CDKN2A | 26 (10.36%) | 137 (17.28%) | 0.009 | (B) Primary |
| FGFR2 | 25 (9.92%) | 144 (18.14%) | 0.0016 | (B) Primary |
| CDKN2B | 20 (9.76%) | 113 (14.87%) | 0.0674 | (B) Primary |
| SMAD4 | 17 (6.77%) | 24 (3.03%) | 0.0138 | (A) Metastasis |
| PIK3CA | 17 (6.75%) | 40 (5.04%) | 0.338 | (A) Metastasis |
| BRAF | 14 (5.56%) | 46 (5.79%) | >0.99 | (B) Primary |
| IDH2 | 13 (5.31%) | 45 (5.75%) | 0.875 | (B) Primary |
| ATM | 13 (5.39%) | 42 (5.35%) | >0.99 | (A) Metastasis |
| KMT2D | 12 (6.56%) | 47 (6.44%) | >0.99 | (A) Metastasis |
| KMT2C | 11 (6.79%) | 37 (6.23%) | 0.856 | (A) Metastasis |
| BICC1 | 10 (3.97%) | 28 (3.53%) | 0.703 | (A) Metastasis |
| MET | 9 (3.64%) | 13 (1.64%) | 0.0736 | (A) Metastasis |
| EGFR | 9 (3.57%) | 15 (1.89%) | 0.145 | (A) Metastasis |
| ERBB2 | 9 (3.57%) | 33 (4.16%) | 0.854 | (B) Primary |

**Supplemental Table 2.** Mutational Frequency in Genes by Race

| **Gene** | **(A) White** | **(B) Asian** | **(C) Black** | **p-Value** | **Most enriched in** |
| --- | --- | --- | --- | --- | --- |
| IDH1 | 160 (20.83%) | 16 (17.78%) | 3 (5.56%) | 0.0215 | (A) White |
| TP53 | 149 (19.43%) | 21 (23.33%) | 16 (29.63%) | 0.153 | (C) Black |
| BAP1 | 147 (19.65%) | 11 (12.94%) | 11 (22.00%) | 0.286 | (C) Black |
| ARID1A | 128 (17.14%) | 13 (15.29%) | 8 (16.00%) | 0.898 | (A) White |
| CDKN2A | 126 (16.43%) | 13 (14.44%) | 8 (15.09%) | 0.869 | (A) White |
| FGFR2 | 124 (16.15%) | 14 (15.56%) | 15 (27.78%) | 0.0823 | (C) Black |
| CDKN2B | 104 (13.92%) | 10 (11.76%) | 6 (12.00%) | 0.811 | (A) White |
| PBRM1 | 103 (14.53%) | 9 (11.39%) | 0 (0.00%) | 0.0156 | (A) White |
| KRAS | 99 (12.89%) | 11 (12.22%) | 4 (7.41%) | 0.498 | (A) White |
| IDH2 | 47 (6.16%) | 2 (2.27%) | 1 (1.89%) | 0.156 | (A) White |
| BRAF | 46 (5.99%) | 7 (7.78%) | 1 (1.85%) | 0.338 | (B) Asian |
| KMT2D | 46 (6.47%) | 5 (6.33%) | 4 (8.70%) | 0.836 | (C) Black |
| ATM | 42 (5.55%) | 3 (3.33%) | 3 (5.88%) | 0.667 | (C) Black |
| PIK3CA | 40 (5.21%) | 2 (2.22%) | 1 (1.85%) | 0.266 | (A) White |
| NF1 | 38 (5.07%) | 3 (3.53%) | 3 (6.00%) | 0.778 | (C) Black |
| RASA1 | 35 (5.66%) | 1 (1.35%) | 2 (5.26%) | 0.288 | (A) White |
| KMT2C | 31 (5.39%) | 6 (9.38%) | 4 (9.76%) | 0.262 | (C) Black |
| MTAP | 31 (12.76%) | 4 (15.38%) | 3 (18.75%) | 0.752 | (C) Black |
| BICC1 | 29 (3.78%) | 2 (2.22%) | 2 (3.70%) | 0.756 | (A) White |
| TERT | 28 (4.03%) | 8 (9.64%) | 3 (6.52%) | 0.0633 | (B) Asian |

**Supplemental Table 3.** Mutational Frequency in Genes by Sex

| **Gene** | **(A) Female** | **(B) Male** | **p-Value** | **Enriched in** |
| --- | --- | --- | --- | --- |
| IDH1 | 124 (23.26%) | 85 (15.98%) | 0.003 | (A) Female |
| BAP1 | 116 (23.43%) | 71 (14.49%) | <0.001 | (A) Female |
| FGFR2 | 112 (21.01%) | 60 (11.28%) | <0.001 | (A) Female |
| TP53 | 97 (18.20%) | 129 (24.29%) | 0.0164 | (B) Male |
| ARID1A | 86 (17.37%) | 84 (17.18%) | >0.99 | (A) Female |
| CDKN2A | 77 (14.45%) | 83 (15.66%) | 0.607 | (B) Male |
| CDKN2B | 62 (12.53%) | 66 (13.50%) | 0.705 | (B) Male |
| PBRM1 | 60 (12.74%) | 65 (14.19%) | 0.564 | (B) Male |
| KRAS | 58 (10.88%) | 77 (14.47%) | 0.0808 | (B) Male |
| IDH2 | 34 (6.48%) | 26 (4.98%) | 0.352 | (A) Female |
| ATM | 30 (5.75%) | 27 (5.16%) | 0.686 | (A) Female |
| KMT2D | 29 (6.16%) | 34 (7.39%) | 0.515 | (B) Male |
| BICC1 | 27 (5.07%) | 11 (2.07%) | 0.0122 | (A) Female |
| BRAF | 25 (4.69%) | 36 (6.77%) | 0.149 | (B) Male |
| PIK3CA | 24 (4.50%) | 33 (6.20%) | 0.224 | (B) Male |
| KMT2C | 23 (6.01%) | 26 (6.72%) | 0.768 | (B) Male |
| RASA1 | 21 (5.12%) | 22 (5.46%) | 0.876 | (B) Male |
| NF1 | 20 (4.01%) | 29 (5.71%) | 0.242 | (B) Male |
| TERT | 16 (3.44%) | 33 (7.17%) | 0.0124 | (B) Male |
| MTAP | 19 (13.10%) | 21 (12.96%) | >0.99 | (A) Female |
